# Regulation and Function of Cdt1; A Key Factor in Cell Proliferation and Genome Stability

**DOI:** 10.3390/genes8010002

**Published:** 2016-12-22

**Authors:** Pedro N. Pozo, Jeanette Gowen Cook

**Affiliations:** 1 Curriculum in Genetics and Molecular Biology, The University of North Carolina at Chapel Hill, Chapel Hill, NC 27599, USA; ppozo@email.unc.edu; 2 Department of Biochemistry and Biophysics, The University of North Carolina at Chapel Hill, Chapel Hill, NC 27599, USA

**Keywords:** cell cycle, DNA replication, genome instability, pre-RC, re-replication, ubiquitylation, cyclin-dependent kinase, geminin, Origin Recognition Complex (ORC), Minichromosome Maintenance (MCM)

## Abstract

Successful cell proliferation requires efficient and precise genome duplication followed by accurate chromosome segregation. The Cdc10-dependent transcript 1 protein (Cdt1) is required for the first step in DNA replication, and in human cells Cdt1 is also required during mitosis. Tight cell cycle controls over Cdt1 abundance and activity are critical to normal development and genome stability. We review here recent advances in elucidating Cdt1 molecular functions in both origin licensing and kinetochore–microtubule attachment, and we describe the current understanding of human Cdt1 regulation.

## 1. Introduction

Origin licensing, the loading of replicative DNA helicases onto origin DNA, is the first committed step of DNA replication and is essential for cell proliferation. Numerous control mechanisms in eukaryotic cells regulate both origin licensing and subsequent replication initiation to ensure complete and precise genome duplication [1,2,3,4,5,6]. Perturbations to origin licensing and replication initiation can result in cell death or in genome instability leading to oncogenesis [1,7,8]. For these reasons, origin licensing control is intimately coordinated with mechanisms that govern cell cycle progression. In this review, we focus specifically on current understanding of the regulation and function of the Cdt1 protein (Cdc10-dependent transcript 1). Unlike other essential licensing proteins, Cdt1 lacks enzymatic activity and shares little resemblance to any other protein of known molecular function, yet it is essential for origin licensing in all eukaryotes tested. In mammalian cells, small changes in Cdt1 control can lead to catastrophic consequences for genome stability, suggesting that Cdt1 regulation is unusually important. Moreover, the recent finding that Cdt1 has a second essential role in the cell cycle during mitosis underscores the importance of fully understanding its function [9]. These features make Cdt1 unique among the core licensing factors and warrant a thorough up-to-date synthesis of the current knowledge about Cdt1 function, structure, regulation, and how its dysregulation contributes to disease. In this review, we focus on understanding mammalian Cdt1, and we are informed by key mechanistic insights gleaned from model experimental systems including *Saccharomyces cerevisiae, Schizosaccharomyces pombe*, *Xenopus laevis*, *Drosophila melanogaster*, and cultured mammalian cells.

## 2. Cdt1 Function

Mammalian cells replicate billions of DNA base pairs with high fidelity and then accurately segregate duplicated genomes to daughter cells each cell cycle. These incredible feats are under strict regulation and are tightly linked to cell cycle progression. Cdt1 is required for both DNA replication and chromosome segregation, and although these functions are not yet fully elucidated, recent advances inspire increasingly detailed models of Cdt1’s role.

### 2.1. Origin Licensing

The first step in eukaryotic DNA replication occurs in G1 and is the sequential loading of replication factors at numerous sites in the genome, known as origins of replication. Origins are sites where DNA replication initiates during S phase. A typical eukaryotic cell contains between 400 (yeasts) and as many as >350,000 (human) potential origins [10,11,12]. Broad distribution of origins on chromosomes ensures complete genome duplication within the time allotted for S phase. Replication factor loading at origins, known as origin licensing, was first described nearly three decades ago using *X. laevis* egg extracts to determine what factors can induce unscheduled DNA re-replication in vitro [13]. The study concluded that DNA replication requires the recruitment of a “Licensing Factor” to DNA during mitosis, thereby setting the stage for DNA synthesis in the subsequent S phase. Furthermore, DNA that was replicated cannot replicate again until the following cell cycle because of the inability of the factor(s) to access chromatin. These results provided the first model for the control of DNA replication where a Licensing Factor binds DNA, is required for the initiation of DNA replication, and becomes deactivated until the following mitosis [13]. Since then, numerous studies have provided experimental support for the now-established “replication licensing system” to control precise genome duplication once-and-only-once per cell cycle [2,14]. The core licensing factors have since been identified, and they assemble into a chromatin-bound macromolecular complex, known as the pre-replication complex (pre-RC). Pre-RC assembly is a highly cell cycle-regulated process governed in part by the cyclical fluctuation of cyclins and the activity of the Cyclin-Dependent Kinases (CDKs) they activate.

The assembly of pre-RCs occurs during a period of low CDK activity in late mitosis and G1 phase. Biochemical and genetic studies in yeast, *Xenopus*, and mammalian cells identified the minimal licensing factors essential for pre-RC assembly [15,16,17,18,19]. These factors are Origin Recognition Complex (ORC), Cell Division Cycle 6 (Cdc6), Minichromosome Maintenance (MCM), and Cdt1. Eukaryotic ORC is a heterohexamer composed of six distinct subunits, Orc1 through Orc6. ORC is the only licensing component that directly binds origin DNA, and it is required for the nucleation of the pre-RC. Cdc6 is a monomeric protein that is recruited to DNA by protein–protein interactions with ORC [16,20,21]. Cdc6 and the Orc1–Orc5 subunits are members of the AAA+ family of ATPases which are prevalent in many DNA metabolic processes [22,23,24]. The MCM complex is the core component of the replicative DNA helicase, and its successful loading onto origin DNA is synonymous with origin licensing. Like ORC, MCM is a heterohexamer composed of six distinct subunits, Mcm2 through Mcm7, which are also AAA+ proteins. In this review, we will specifically discuss Cdt1 regulation and function; for in-depth reviews of ORC, Cdc6, and MCM, the reader is referred to excellent contributions by others in the field [14,23,24,25].

**Figure 1:**
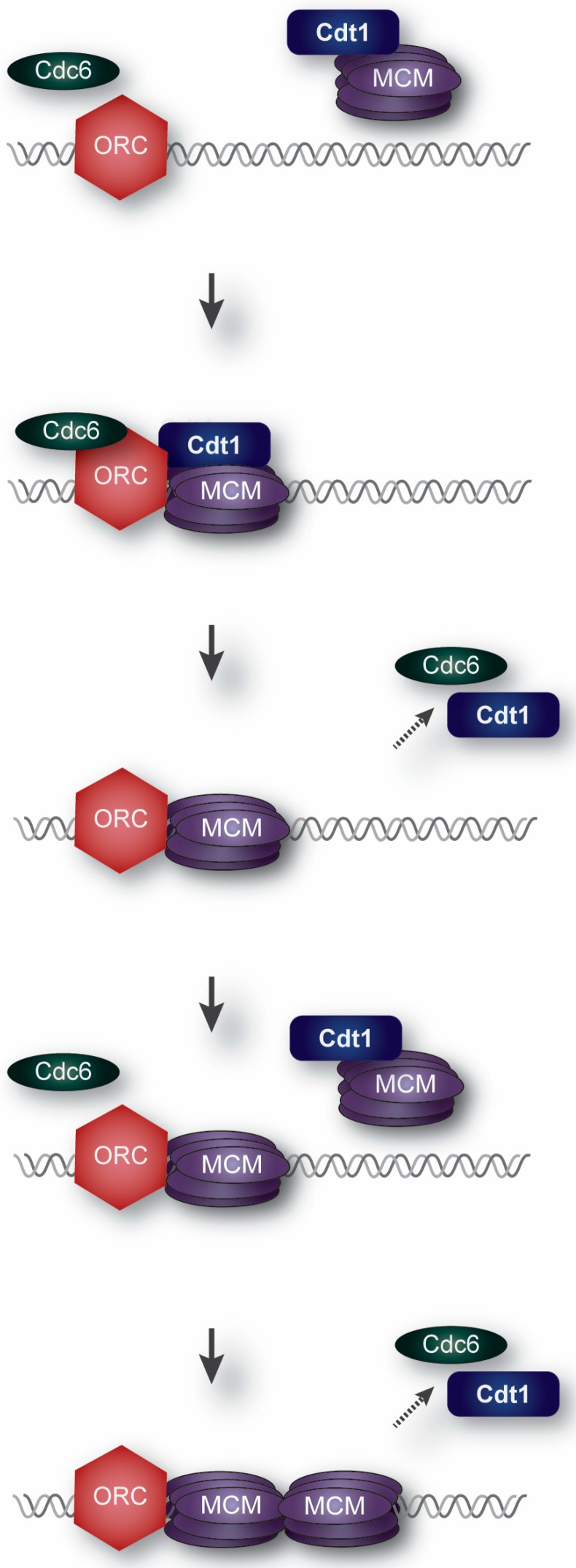
Origin Licensing. Minichromosome Maintenance (MCM) hexamers are loaded by Cdt1, Cdc6, and Origin Recognition Complex (ORC) at presumptive chromosomal origins during G1 phase.

Our understanding of the molecular events in origin licensing (illustrated in Figure 1) comes primarily from pioneering work using both *X. laevis* egg extracts and purified budding yeast licensing proteins [2,5]. Importantly, the strong conservation of origin licensing proteins throughout eukaryotic evolution, combined with many corroborating studies in mammalian cells, gives confidence that licensing functions elucidated in model systems are applicable to human cells; though aspects of their regulation vary by species. Pre-RC assembly begins with ORC loading onto presumptive origin DNA. Interestingly, ORC DNA binding—particularly in metazoan genomes—is largely independent of DNA sequence, but is highly influenced by local chromatin characteristics [26,27,28]. ORC recruits the Cdc6 protein to chromatin to await the arrival of Cdt1 bound to the MCM complex to form a pre-RC [2,5]. In a process not yet fully understood [29,30], the concerted action of ORC, Cdc6 and Cdt1 results in topological loading of an MCM heterohexamer onto DNA with double-stranded DNA passing through the MCM central channel [18,19]. Cdc6 and then Cdt1 are released, followed by a second round of Cdc6 and Cdt1-MCM recruitment [31]. The second MCM complex is loaded such that the MCM N-termini face one another to create double hexameric rings. This arrangement sets each MCM complex in the correct orientation to establish bidirectional forks upon origin firing [32,33]. Only the correct loading of MCM double hexamers renders a locus competent for subsequent replication initiation, or “firing”, during S phase. MCM loaded in G1 is not active as a helicase, and origin DNA is thought to remain double-stranded until origin firing. Origin firing requires phosphorylation events from CDKs and a replication-specific kinase, Dbf4-dependent kinase (DDK). These kinases promote the recruitment of additional essential helicase components, Cdc45 and GINS, to activate DNA unwinding [34,35,36].

Origin licensing can begin as early as telophase, as soon as nuclear envelopes have formed around the segregated mitotic chromosomes, though it is not clear if licensing begins this early in all species or cell types [37,38,39]. Licensing continues throughout G1 and ceases at the G1/S phase transition. Somewhat surprisingly, eukaryotic cells load many more MCM double hexamers than the number of DNA-bound ORCs [40]. At least 10-fold more origins can be licensed than are strictly required for complete replication under normal circumstances, though the degree of origin licensing likely varies among cells, tissues, and species [41,42,43]. In vitro, loaded MCM double hexamers can slide along DNA away from ORC, leaving space near ORC for another round of MCM loading [18,19], and recent results suggest that MCMs may also slide in vivo [42,44]. In a typical S phase, some MCM complexes that had been loaded in G1 are activated as part of the regular replication program, whereas others initiate replication in response to nearby stalled or damaged replication forks to ensure replication completion. Origins that are only utilized under the latter conditions of replication stress are termed “dormant” origins, and they safeguard the genome against under-replication. [45,46,47].

Notably, Cdt1 is essential for MCM loading in all eukaryotes in which it has been tested, but its precise molecular function in origin licensing is not fully clear [48,49,50]. Cdt1 interacts directly with the MCM complex in solution and with both ORC and Cdc6 [51,52,53,54,55]. In the absence of Cdt1, MCM complexes are never recruited to DNA [48,56,57]. In that regard, one likely role for Cdt1 in licensing is as a molecular bridge or “courier” to deliver soluble MCM complexes to DNA-bound ORC/Cdc6. In support of that model, recent single molecule studies using purified yeast licensing proteins discovered that Cdt1 is rapidly released upon successful loading of each MCM complex [31]. By following individual labeled proteins, Ticau et al. showed Cdt1 and Cdc6 release between the two rounds of MCM loading. This rapid shuttling between the bound and soluble states for both Cdt1 and Cdc6 suggests that each molecule could participate in many origin licensing events. Perhaps for this reason, the levels of both Cdc6 and Cdt1 are highly regulated during the cell cycle to prevent inappropriate origin licensing.

The MCM complex is a hexameric ring even in solution before it is loaded [18,36,58]. MCM loading is therefore not a process of assembling the heterohexamers on DNA from their component subunits, but rather, loading pre-assembled hexamers onto DNA. DNA passes through a side “gate” between the Mcm2 and Mcm5 subunits, and much speculation currently swirls around the mechanism and dynamics of MCM gate opening and closing [59,60]. Moreover, the MCM double hexamer central channels contain double-stranded DNA in G1 but the active MCM helicase at replication forks encircles single-stranded DNA and displaces the second strand [35,61,62]. At least in vitro, yeast Cdt1 is not released from the complex until MCM is successfully loaded [31]. Its persistence during the actual loading reaction suggests that Cdt1 does more than simply hand MCM off to ORC and Cdc6. Cdt1 may be required to maintain MCM in the proper orientation or conformation for successful DNA loading. If so, then how Cdt1—or ORC and Cdc6 for that matter—load the two MCM complexes in opposite orientations remains to be discovered [14,30].

### 2.2. De-Regulated Origin Licensing

The requirement that normal DNA replication produce exactly one copy of each chromosome puts important constraints on origin utilization. Specifically, each origin that fires, must fire no more than once per cell cycle. Origin re-firing results from re-licensing DNA that has already been duplicated. A second round of initiation from the re-licensed origins leads to duplicating sequences more than once, a phenomenon known as re-replication. Interestingly, re-replication is induced in the final cell cycles of some tissues to increase DNA copy number, most notably in *D. melanogaster*, but such cells are not normally destined to divide again. Re-replication is distinct from scheduled genome re-duplication which results from skipping cytokinesis; re-duplication typically produces quantile increases in ploidy whereas developmentally programmed re-replication targets only some loci [63,64,65]. In contrast to developmentally programmed re-replication, unscheduled re-replication is an aberrant phenomenon associated with genome instability [3,6]. Indeed, re-replication can be the initiating event for gene amplification [66], a frequent observation in cancer cells. Partial re-replication can be experimentally induced by deregulating MCM loading factors, and in human cells, re-replicated sequences are detectable essentially randomly throughout the genome [67]. Re-replication is typically associated with markers of replication stress and evidence of DNA damage response pathway activation [68,69,70,71].

To avoid re-replication, all origin licensing activity ends once S phase begins. There is no known means to directly reverse inappropriate origin licensing, so a network of overlapping inhibitory mechanisms is needed to prevent all origin licensing outside of G1 phase. These licensing controls target each member of the pre-RC from the onset of S phase through mitosis. Mammalian Cdt1 is inhibited by at least four distinct pathways, suggesting that it is among the most important to inhibit; we discuss each of these mechanisms in more detail in Section 4. Many licensing factors are inactivated by phosphorylation via the same CDK activity that triggers origin firing (in human cells primarily cyclin A/Cdk2). Interestingly, the outcomes of these phosphorylations may vary depending on the licensing factor being targeted and in which organism, though the end result is always to inhibit origin relicensing. For example, in *S. cerevisiae*, Cdc6 phosphorylation by CDK targets it for ubiquitin-mediated proteolysis, whereas phosphorylation of human and *Xenopus* Cdc6 induces nuclear export [20,72,73,74]. On the other hand, in *S. cerevisiae,* MCM and Cdt1 are subjected to CDK-mediated nuclear export [56,75]. In *S. cerevisiae,* ORC subunits are inhibited by CDK-dependent phosphorylation by disrupting their ATPase activity [76] and blocking interaction with Cdt1 [77], whereas in human and *Xenopus*, CDK-dependent ORC phosphorylation induces release from origins and/or degradation of the Orc1 subunit [78,79,80]. Regardless of the species-specific details, the aggregate result is inhibiting pre-RC assembly by neutralizing interactions or triggering licensing factor degradation.

Incomplete origin licensing in G1 can also be a source of genome instability. In untransformed human cells, significantly slowing origin licensing induces a delay in S phase onset by delaying the activation of Cdk2 [81,82,83]. This “origin licensing checkpoint” requires p53, meaning that p53-deficient cells can enter S phase with severely underlicensed chromosomes which renders them susceptible to S phase failure [81,82,83]. Despite extensive documentation of the licensing checkpoint phenomenon in several labs and in multiple cell lines, precisely how licensed or unlicensed DNA is detected to affect Cdk2 activity is still unclear. Moreover, “sufficient” origin licensing is not simply a matter of the total number of loaded MCM hexamers per genome since their distribution is also critical. A recent study by Moreno et al. found that moderate licensing inhibition that does not cause a cell cycle delay, nonetheless increases the likelihood that regions of unreplicated DNA persist through mitosis [84]. Thus, Cdt1 activity and origin licensing must be efficiently blocked in S phase and G2 to prevent re-replication but must be fully induced in G1 to ensure sufficient origin licensing and complete genome duplication.

### 2.3. Cdt1-Associated Chromatin Modifiers

Licensing factors must have local access to origin DNA to assemble and load MCM helicases. The chromatin environment at origins thus has a large impact on origin licensing. Post-translational histone modifications, such as methylation and acetylation, can greatly affect DNA accessibility which may facilitate ORC binding, MCM loading, and/or origin firing. In addition, at *S. cerevisiae* origins which have been mapped with high resolution, nucleosome positioning also plays a role in determining ORC localization and activity (reviewed in [10,27,85]). In the majority of eukaryotic genomes, DNA sequence is a minor determinant of origin location. The model that has emerged is that ORC is recruited to DNA not by a specific nucleotide sequence, but rather by aspects of local chromatin structure and DNA accessibility. Some evidence supporting this model is the presence of a BAH (Bromo Adjacent Homology) domain in Orc1, the largest subunit of ORC. The BAH domain specifically recognizes histone post-translational modifications (PTMs) enriched at replication origins, and is required for proper ORC DNA loading [86,87].

Once ORC has bound, the local chromatin environment may require additional modifications to permit efficient origin licensing. Several histone-modifying enzymes associate with licensing components and are predicted to modify nucleosomes to promote DNA accessibility; some of these enzymes have been identified as Cdt1 partners. One such chromatin modifier is histone acetyltransferase bound to Orc1 (Hbo1), which as its name implies, was first discovered as an Orc1-binding protein and later shown to bind the Mcm2 subunit of MCM, and Cdt1 [88,89,90]. Hbo1 is highly conserved, and orthologs in *D. melanogaster* and *S. cerevisiae* have also been linked to DNA replication [91,92]. In human cells, Hbo1 is responsible for the bulk of histone H4 acetylation genome-wide [93]. Since histone H4 acetylation generally correlates with active chromatin and accessible DNA, increased local histone acetylation could promote origin licensing. In addition, Hbo1 was specifically detected at several known human replication origins during G1 coincident with Cdt1 origin association [90]. Further studies found that Cdt1 promoted chromatin openness in association with Hbo1 during G1, likely increasing local chromatin accessibility and facilitating MCM loading [94]. In addition to Hbo1, early proteomic screens for Cdt1-interacting proteins discovered the GRWD1 protein (glutamate-rich WD40 repeat containing 1), a histone binding protein [95]. Follow up studies suggested that GRWD1 regulates chromatin openness during MCM loading at replication origins [95] and may cooperate with a chromatin remodeler, SNF2H [96]. On the other hand, during S phase and G2 Cdt1 may contribute to inhibiting origin licensing by recruiting the HDAC11 histone deacetylase. Local histone deacetylation would presumably reduce chromatin accessibility and inhibit origin relicensing [94,97]. Interestingly, association of the inhibitor protein geminin with Cdt1 during S phase enhanced the recruitment of HDAC11 to origins to further inhibit origin licensing [94].

### 2.4. Cdt1 in Chromosome Segregation

Surprisingly, human Cdt1 is required not only for origin licensing but also for mitosis. As a consequence, asynchronously-growing cells, depleted of Cdt1, accumulate in both G1 phase and G2 phase because they can neither license origins, nor progress through the metaphase-to-anaphase transition. This essential mitotic function was first discovered in a screen for Cdt1-interacting proteins that identified human Hec1 (Highly Expressed in Cancer 1), a component of the NDC80 kinetochore–microtubule attachment complex [9]. Hec1 is conserved from yeast to mammals, but the mitotic Cdt1 function is not evident in either budding or fission yeast [57,98]; more work is required to determine if Cdt1 has mitotic functions in invertebrates such as *D. melanogaster* or *Caenorhabditis. elegans* or in non-mammalian vertebrates such as *X. laevis*.

A fraction of human Cdt1 molecules localize to kinetochores in mitosis, and this localization requires Hec1; Hec1 localization is unaffected by Cdt1 depletion. Cdt1 interacts with and is recruited to kinetochores via a unique “loop” domain in Hec1 that interrupts an otherwise long coiled-coil central span. Both depletion of Cdt1 prior to mitosis or mutationally altering the Hec1 loop domain to block Cdt1 binding and recruitment resulted in prometaphase arrest with an unsatisfied spindle assembly checkpoint [9]. Importantly, the mitotic defect in Cdt1-depleted cells can be separated from potential indirect effects of incomplete DNA replication by depleting Cdt1 after origin licensing is complete and S phase has already begun [9].

It is not yet clear precisely how Cdt1 promotes stable kinetochore–microtubule attachments since it is not required for the localization of any other kinetochore proteins tested thus far. One clue to its function came from analysis of the conformation of the NDC80 complex in vivo using super-resolution microscopy. The structure of the NDC80 complex (Hec1/Nuf2/Spc24/Spc25) indicates that the loop region of Hec1 where Cdt1 binds is a point of flexibility in an otherwise long and rigid coiled-coil domain. Prior work by Wang et al. supported the notion that the loop region corresponds to a hinge or joint in the complex [99]. The N-terminal domains of Hec1 and Nuf2 directly contact kinetochore microtubules, whereas the Spc24 and Spc25 subunits connect the complex to other kinetochore proteins [100,101]. In prometaphase, prior to attachment, the two ends of the NDC80 complex are relatively close together, whereas at stably-attached kinetochores in metaphase, the two ends of the complex are considerably further apart [101]. Mutation of the loop domain or depletion of Cdt1 prevented this extended NDC80 conformation [9]. Thus, Cdt1 supports a microtubule-dependent conformational extension in its partner, the NDC80 complex, by interaction with the major point of flexibility conferred by the loop region of Hec1.

Many important questions about Cdt1 mitotic function remain: what other (if any) microtubule-associated or kinetochore partners bind Cdt1? The Hec1-interacting domain on Cdt1 is not yet known, but identifying this region is a first step towards generating separation-of-function alleles that are impaired for only origin licensing or only kinetochore–microtubule attachment. How, precisely, does Cdt1 affect the conformation of NDC80? Moreover, as described below (see Section 4.4), Cdt1 is heavily phosphorylated during G2 phase and mitosis. What role does Cdt1 phosphorylation play in its intermolecular interactions and function at kinetochores? Clearly, much remains to be learned about this novel role for Cdt1 and how it relates to the more famous origin licensing function.

## 3. Cdt1 Structure

In most species, Cdt1 is a ~60–70 kDa protein; *S. pombe* Cdt1 is somewhat smaller at ~50 kDa whereas the *D. melanogaster* Cdt1 is ~82 kDa. (*D. melanogaster* Cdt1 is named “double-parked”, abbreviated Dup, but nearly all other species use “Cdt1” as the protein and gene name). Although each subunit of ORC and MCM, Cdc6 and Cdt1 are conserved in all eukaryotic genomes examined, the degree of sequence conservation is lowest for Cdt1 compared to the other licensing proteins. Indeed, the low sequence similarity between human and *S. cerevisiae* Cdt1 coupled with the unusual history of metazoan Cdt1 being identified first, led to a brief period in the field when it was not clear if budding yeast had a Cdt1 ortholog. Focused sequence searches coupled with functional tests ultimately identified the yeast Cdt1 ortholog [57]. Unlike nearly all other licensing components which are homologous to AAA+ ATPases, Cdt1 is not an enzyme, and the Cdt1 protein sequence bears little similarity to other proteins of known molecular activity. Although the Cdt1 sequence gives little insight into its function, some information about interacting regions, post-translational modifications, and domain structures is available which we describe here.

### 3.1. Functional Domains

**Figure 2:**
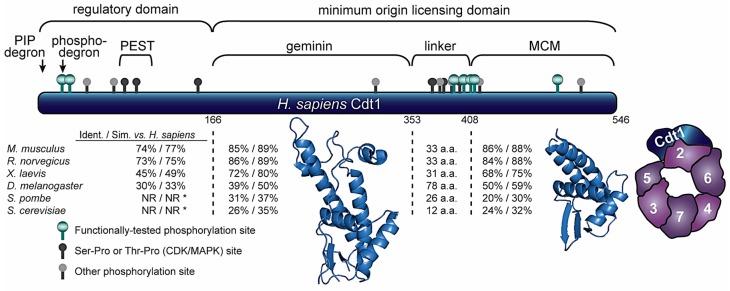
Human Cdt1 structure. Diagram of Cdt1 divided into four segments based on alignments and structural studies. Pairwise comparisons to the human sequence for representative eukaryotic Cdt1 orthologs within each segment are reported as % identity/% similarity; NR indicates regions in fungal sequences too short or dissimilar for comparison. Regions responsible for recognition by E3 ubiquitin ligases (degrons), a region enriched in proline, glutamic acid, serine, and threonines (PEST domain), geminin binding, MCM) binding, and a putative linker domain (enriched in phosphorylation sites) are marked. Phosphorylation sites in human Cdt1 that are conserved in at least one other vertebrate sequence are marked as ball-and-stick icons: green = Cyclin-Dependent Kinases (CDK)/Mitogen-Activated Protein Kinases (MAPK) sites validated by mutagenesis and functional studies, dark gray = putative CDK/MAPK sites (serine-proline or threonine-proline) identified by mass spectrometry [104], light gray = conserved sites detected by mass spectrometry distinct from the CDK/MAPK substrate consensus. Ribbon diagrams of the two segments for which structures have been determined are shown; central domain PDB 2WVR (human) and C-terminal domain PDB 3A4C (mouse) [105,106]. A diagram of the yeast MCM2-7 complex bound to full-length Cdt1 derived from tracing the single-particle analysis results from Sun et al. 2013 is also shown.

Multispecies Cdt1 protein sequence alignments reveal regions that are relatively well-conserved and regions which share considerably less conservation. Not surprisingly, the regions of low conservation are particularly prominent in comparisons of mammalian and fungal Cdt1 species. Using human Cdt1 as a reference, Figure 2 includes pairwise sequence comparisons between human Cdt1 and Cdt1 sequences from several model organisms in four Cdt1 domains, the N-terminus (amino acids [aa] 1–166), the central domain (aa 167–374), a short “linker” region (aa 375–406), and the MCM binding domain (aa 407–546). Sites of protein–protein interactions and phosphorylations are also marked. The N-terminal sequences of both model yeast Cdt1 sequences are generally quite short and they bear little resemblance relative to their metazoan counterparts. On the other hand, sections of higher relative homology suggest regions important for functions that are conserved in all species, such as interaction with other origin licensing components.

Traditional truncation and mutagenesis approaches identified Cdt1 domains required for protein interactions and for specific aspects of origin licensing function [54,102,103]. The most comprehensive of these studies by Ferenbach et al. validated and/or delineated the MCM binding domain, geminin binding domain, and minimal licensing activity domain using recombinant fragments of *X. laevis* Cdt1 added to oocyte lysates. The shortest fragment that complemented Cdt1-depleted lysates for licensing corresponds to human Cdt1 aa 243–546 [54]. The finding that the N-terminal 242 amino acids (corresponding to human aa 1–170) are dispensable for licensing activity, plus the fact that this region is the least-well-conserved is consistent with the notion that the N-terminal region is the target of species-specific regulation rather than essential for Cdt1 function.

### 3.2. Crystal Structures/Cryo-EM Structures

Currently, no atomic structure for full-length Cdt1 from any species is available. One challenge for structure studies of Cdt1 is that both the N-terminal domain and part of the linker domain are predicted to be intrinsically disordered. Using two different prediction tools, the N-terminal 166 amino acids of human Cdt1 has a probability of disorder at each position greater than 65% [107,108]. The linker is relatively short, but it also contains a region of high predicted disorder. Trimming these regions to isolate the central domain or the C-terminal domain yielded fragments that were compatible with crystallography, and their exclusion from the structural studies is consistent with the notion that they are flexible. The atomic structure of the central domain was first solved for mouse Cdt1 (aa 172–368) in complex with the geminin inhibitor protein [105], and the corresponding human Cdt1 protein fragment (aa 166–353) was later crystallized [106]. A recent search of a database of protein structures for nearest neighbors to this central domain identified some similarity to winged-helix domains [109]. Otherwise, the central domain structure is relatively unique.

The C-terminal domain (human 408–546) interacts with the MCM complex. This isolated fragment can directly bind a C-terminal fragment from the Mcm6 subunit suggesting that this interaction is one of the direct contacts between Cdt1 and the MCM complex in vivo [110]. This protein fragment was characterized by both X-ray crystallography and Nuclear Magnetic Resonance as a winged helix domain [102,103,111]. Interestingly, this winged-helix shows some structural similarity to the central domain of Cdt1 itself [102]. Although winged helices are most well-known for roles in nucleic acid binding, the C-terminal Cdt1 winged-helix is unlikely to form stable interactions with DNA. Positions of key alpha helices are incompatible with DNA binding compared to winged-helix domains in canonical DNA binding proteins, and Cdt1 lacks charged patches that would stabilize DNA binding [102]. Moreover, Cdt1 chromatin association in cells requires ORC [48,77], and purified yeast Cdt1 does not bind origin DNA in the absence of ORC [19]. It is most likely therefore that the C-terminal Cdt1 winged-helix is of the type that mediates protein–protein rather than protein-nucleic acid interactions. In support of that model, mutational alteration of a subset of charged surface residues of the C-terminal domain impaired MCM binding in vitro [102,111], and several of the corresponding mutations to budding yeast Cdt1 impaired cell growth [102]. These biochemical data corroborated findings from a separate co-crystallographic study which demonstrated a direct Cdt1–Mcm6 interaction conferred by the Cdt1 C-terminal domain [103].

Although these studies provide important structural information, key aspects of Cdt1 structure are still not known. Yeast Cdt1 can directly bind the Orc6 subunit of ORC [55], but the Cdt1 domain responsible is not known nor are potential Cdt1 regions that bind other subunits of MCM. As-yet uncharacterized Cdt1 interactions with ORC and MCM are likely required for origin licensing and/or regulating Cdt1 function. In that regard, a recent paper described a novel and still uncharacterized “PEST” (rich in proline [P], glutamic acid [E], serine [S], and threonine [T]) domain in mouse Cdt1 (Figure 2) [112]. Cdt1 is abundant during G2 phase but is poorly associated with chromatin [38,112,113]. Truncating the PEST domain caused premature re-association of Cdt1 with chromatin during G2 and increased the likelihood of re-replication [112]. Given that Cdt1 chromatin binding requires ORC interaction [48], this PEST domain may indicate a region required for ORC binding.

Several studies using single particle electron microscopy coupled with labeling strategies have suggested how full-length budding yeast Cdt1 interacts with the MCM complex and in a licensing intermediate containing ORC, Cdc6, Cdt1 and MCM [29,32]. These models are consistent with the biochemical studies detecting Cdt1 in direct contact with Mcm6 [77]. In addition to this contact with Mcm6, Cdt1 appears to contact additional MCM subunits, especially extensive interaction with Mcm2 (Figure 2). This location is relatively close to the interface of Mcm2 with Mcm5 through which DNA passes during the loading reaction [59,60]. In this position, Cdt1 is well-placed to affect the conformation of the MCM complex during loading in ways that may stabilize either the open or closed MCM conformation.

## 4. Cdt1 Regulation

**Figure 3:**
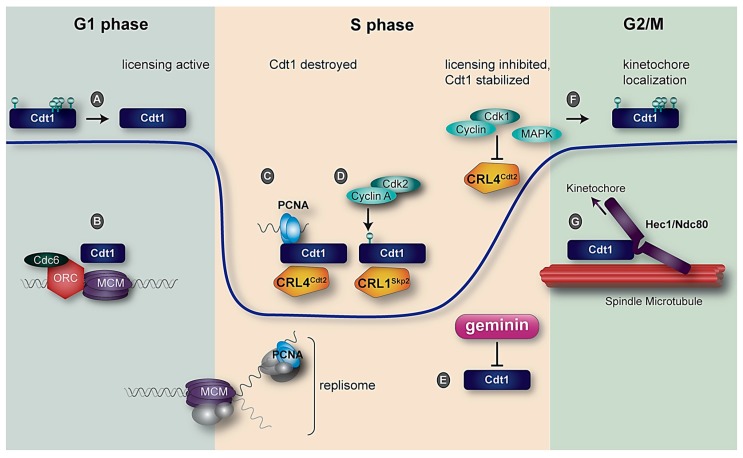
Human Cdt1 regulation during a single cell cycle. The blue line indicates relative Cdt1 protein abundance. (**A**). Cdt1 is dephosphorylated in early G1 by an unknown phosphatase; (**B**) Cdt1 participates with ORC and Cdc6 to load MCM hexamers onto DNA; (**C**) Proliferating Cell Nuclear Antigen (PCNA) loaded at DNA replication forks is bound by the Cdt1 PCNA-Interacting Protein (PIP) degron, and the complex is recognized for ubiquitylation and subsequent proteasome-mediated destruction by CRL4^Cdt2^; (**D**) Cdt1 is phosphorylated at Thr29 by cyclin A/Cdk2 to create a phosphodegron recognized for ubiquitylation by CRL1^Skp2^. The combined action of two E3 ubiquitin ligases drives Cdt1 degradation in S phase; (**E**) The geminin protein begins to accumulate in early S phase, and peaks in late S phase and G2. Geminin binding blocks Cdt1 origin licensing function; (**F**) During late S phase and G2, mitotic kinases—especially cyclin A/Cdk1 and the stress-activated MAP kinases p38 and c-Jun N-terminal Kinase (JNK)—phosphorylate Cdt1; Cdk1 also inactivates CRL4^Cdt2^; (**G**) A subset of Cdt1 molecules is recruited to kinetochores in mitosis through interaction with the loop domain of Hec1. Cdt1 is required for stable kinetochore–microtubule attachment.

To properly license origins for DNA replication in G1 and block origin licensing from the onset of S phase through mitosis, multiple independent mechanisms control human Cdt1 abundance and function (illustrated in Figure 3). Although other licensing proteins are also under cell cycle control, Cdt1 is subject to the most extensive regulation in human cells, suggesting that it is perhaps the most important licensing factor to regulate in mammalian cells. The ultimate outcome is a collection of cell cycle-dependent regulatory mechanisms that allow Cdt1 to function efficiently in its origin licensing role during G1, prevent origin relicensing in S and G2 phase, and permit Cdt1 participation in kinetochore microtubule attachment during mitosis.

### 4.1. Transcriptional Control

The first Cdt1 ortholog was cloned 20 years ago in a screen for fission yeast transcripts that are upregulated at the G1 to S transition [98]. In fission yeast, the transcription factor driving Cdt1 expression is Cell Division Cycle 10 (Cdc10), which is responsible for transcriptional induction of many genes important for the G1 to S phase transition [114]. The analogous function in metazoans is the responsibility of the E2F family of transcription factors, though the protein sequences of Cdc10 and E2F themselves are unrelated [115,116]. The human *CDT1* gene has three putative E2F responsive elements in its promoter region, is activated by E2F with peak expression in late G1, and is inhibited by the Rb tumor suppressor [117]. Other studies have suggested that Cdt1 is also under the transcriptional control of the c-*Myc* proto-oncoprotein and the Gli1 component of the hedgehog signaling pathway [118,119]. Of note, Cdt1 protein abundance in proliferating cells peaks in G1 and G2 rather than S phase (Figure 3), but the transcriptional upregulation generally supports Cdt1 expression during proliferation. Aside from the documented regulation by E2F and possibly c-myc and Gli1, little else is known about how the production of Cdt1 is regulated. For instance, no evidence for alternative splicing, regulation by microRNAs or translational control has yet emerged, though such possibilities should be explored.

### 4.2. Ubiquitin Mediated Proteolysis in S Phase

A key aspect of re-replication control in metazoans is ubiquitin-mediated Cdt1 degradation during S phase. This regulation occurs in all eukaryotes except for budding yeast in which a Cdt1-MCM complex is exported from the nucleus to the cytoplasm during S phase [56,75]. In mammalian cells, Cdt1 degradation in S phase is mediated by two independent E3 ubiquitin ligase complexes, CRL1^Skp2^ (also known as SCF; reviewed in [120]) and CRL4^Cdt2^ [121,122]. Like many substrates of CRL1^Skp2^, Cdt1 is only bound for productive ubiquitylation once it is phosphorylated by CDK. In human Cdt1, this “phosphodegron” is created by Cdk2-mediated phosphorylation at threonine 29. Thr29 phosphorylation is then recognized by Skp2, a substrate adaptor, to trigger ubiquitylation [123,124,125]. A nearby serine at position 31 that matches the minimal CDK substrate consensus is also phosphorylated in cells, but it plays a minor role in recruiting Cdt1 to CRL1^Skp2^.

Although manipulations that block Cdt1 Thr29 phosphorylation prevent ubiquitylation by CRL1^Skp2^, such manipulations do not substantially stabilize Cdt1 during S phase. Even in the absence of CRL1^Skp2^ targeting, Cdt1 is ubiquitylated and degraded by a second E3 ubiquitin ligase, CRL4^Cdt2^ [126] (Cdt2 was identified in the same screen that discovered Cdt1, but the Cdt1 and Cdt2 sequences are unrelated [98]). Unlike targeting by CRL1^Skp2^, Cdt1 ubiquitylation by CRL4^Cdt2^ is not stimulated by Cdt1 phosphorylation, but instead requires a ternary interaction among Cdt1, the substrate adapter Cdt2, and DNA-loaded Proliferating Cell Nuclear Antigen (PCNA). PCNA is a homotrimer that is loaded by Replication Factor C at replication forks and serves as the processivity factor for DNA polymerase during DNA replication. DNA-bound PCNA is also a platform for a host of proteins that bind PCNA through short linear motifs known as PCNA-Interacting Protein (PIP) boxes [127]. The Cdt1 PIP box is special in that it not only binds PCNA but also triggers degradation and is thus termed a “PIP degron.” PCNA is only loaded during DNA synthesis, and this loading event is required for Cdt1 recognition by CRL4^Cdt2^; thus this mode of Cdt1 degradation has been termed “replication-coupled destruction” [127]. Since the trigger for CRL4^Cdt2^-mediated degradation is PCNA DNA loading, PIP degron-containing Cdt1 proteins are also degraded after DNA damage because PCNA is loaded during DNA repair [128].

Mutations to the human Cdt1 PIP degron alone have only modest effects on S phase degradation in otherwise unperturbed cells. On the other hand, a combination of PIP degron mutations with mutations that block Cdt1 phosphorylation at Thr29 stabilizes Cdt1 during S phase and induces substantial re-replication [129]. Near the end of S phase, human Cdt1 re-accumulates, but this re-accumulation is not strictly because PCNA is no longer DNA loaded. CRL4^Cdt2^ is globally inhibited as cells approach G2, leading to re-accumulation of all of its substrates [130]. Cdt1 is clearly not targeted by CRL1^Skp2^ in G2 phase either, although cyclin A-dependent activity is still high. The mechanism preventing CRL1^Skp2^-mediated Cdt1 degradation in G2 is still unknown. One potential addition to Cdt1 stability control is the recent report that Cdt1 abundance is sensitive to the deubiquitylating enzyme, Usp37 [131]. Thus, Cdt1 re-accumulation could be as much a consequence of increased deubiquitylation as it is a result of decreased ubiquitylation.

Somewhat surprisingly and despite being a particularly potent inducer of S phase destruction [132], the PIP degron is not conserved in all Cdt1 proteins—not even among all mammalian Cdt1 sequences. PIP degron sequences are not evident in the cow, pig, sheep, or rabbit Cdt1 sequences, though Cdt1 PIP degrons are found in nematode, fruit fly, zebrafish, chicken, rat, mouse, baboon and many other species (J.G.C. unpublished observation and [121]). Moreover, CRL1^Skp2^-mediated degradation to reinforce CRL4^Cdt2^-mediated degradation may not be universal among metazoans (e.g., *X. laevis* Cdt1). In species where it appears that only one E3 ligase targets Cdt1 during S phase, the presence of stronger licensing inhibitory mechanisms that target other pre-RC components may have allowed the second E3 pathway to be lost.

### 4.3. Inhibition by Geminin

Unlike nearly all components of the replication licensing system, human Cdt1 was not cloned strictly on the basis of sequence homology to a yeast ortholog. In fact, the fission yeast Cdt1 was not directly investigated as a licensing protein until after the metazoan Cdt1 proteins were functionally characterized. Human Cdt1 was isolated both by sequence similarity to *D. melanogaster* and *X. laevis* orthologs and as the target of a re-replication inhibitor protein, geminin [48,133,134]. Geminin itself was cloned from biochemical screens for *X. laevis* proteins that are degraded in mitosis [135]. Of note, neither budding nor fission yeast harbor a geminin ortholog. Human geminin is abundant during S phase and G2, is degraded at anaphase, and is least abundant during G1 phase. Geminin is a substrate of the APC/C (Anaphase-Promoting Complex/Cyclosome) [135], an E3 ubiquitin ligase which promotes geminin degradation from late mitosis and throughout G1 phase [136,137].

Artificially elevating geminin concentration in G1 blocks MCM loading, but the mechanism of that inhibition was not known at the time geminin was first characterized [135]. An effort to gain insight into how geminin inhibits licensing by identifying partners yielded a tight-binding partner in human lysates, human Cdt1. Moreover, supplementing geminin-inhibited *X. laevis* lysates with additional human Cdt1 reversed the inhibitory effects of geminin on origin licensing and DNA replication [134]. Mutations in Cdt1 that alter geminin binding also have higher licensing activity in vitro compared to wild-type Cdt1 [138]. Like Cdt1, geminin has at least one alternative function outside the licensing system; geminin regulates gene expression during development [139,140,141].

Geminin is a dimer that forms a stable 2:1 complex with monomeric Cdt1 both on chromatin and in the nucleoplasm. Geminin binds the central region of Cdt1 (Figure 2), and indeed both high-resolution structures of this Cdt1 domain are in complex with geminin; possibly because the tight binding facilitated crystallization [105,106]. Interestingly, the human crystal structure consists of a Cdt1 and geminin heterohexamer composed of two Cdt1 and four geminin polypeptides; this structure is essentially a dimer of the trimer observed in the mouse structure. Based on this and other observations, De Marco et al. suggested that the trimer is permissive for licensing, whereas the hexamer corresponds to the inhibited form [106]. If true, then only high concentrations of geminin, such as those found in mid-to-late S phase and G2, would be effective for forming hexamers and preventing re-replication. In this scenario, re-replication control in early S phase should rely more heavily on Cdt1 degradation and mechanisms that target ORC, Cdc6, and MCM than on geminin because geminin is less abundant in early S phase.

How does geminin inhibit Cdt1 activity? In vitro, geminin prevents the association of Cdt1 with MCM complexes [51,142] and also blocks Cdt1 binding to Cdc6 [51]. A simple stearic occlusion model seems unlikely however, if the primary binding site for MCM is the Cdt1 C-terminal domain but geminin binds the central domain. Nonetheless, geminin dimers bound to the Cdt1 central domain could conceivably project far enough towards the C-terminal domain to interfere with stable MCM binding. Alternatively, geminin binding may induce a conformational change in the Cdt1 central domain that propagates to the C-terminal domain. It may also be that Cdt1 forms multiple contacts with the MCM ring, and that geminin interferes with MCM binding sites in Cdt1 that are separate from those at the C-terminal Cdt1–Mcm6 interface (e.g., the diagram based on Sun et al. 2013 in Figure 2). Testing these ideas directly will ultimately require a structure including both the Cdt1 central and C-terminal domains with and without geminin.

### 4.4. Cdt1 Phosphorylation

Cdt1 is phosphorylated at many serine and threonine (but not tyrosine) sites at different times during the cell cycle and in response to different cues. Phosphoproteomic analyses have identified dozens of phosphorylation sites detectable in proliferating human cells. Figure 2 marks only those human Cdt1 phosphorylation sites from the PhosphoSite Plus database [104] that are conserved in at least one other mammalian Cdt1. Those marked in dark grey are Ser-Pro or Thr-Pro sites which conform to the minimal recognition sequence for both CDKs and MAP kinases (mitogen-activated protein kinases). The green symbols mark Ser-Pro and Thr-Pro sites that have been identified in proteomics screens and also tested for functional consequences.

As discussed above (Section 4.2), phosphorylation at Thr29 targets Cdt1 for ubiquitylation by the CRL1^Skp2^ E3 ubiquitin ligase. Which kinase (or kinases) is most responsible for Thr29 phosphorylation? In vitro, Cdt1 can be phosphorylated by Cdk4 (activated in G1 by cyclin D), Cdk2 (activated in S by cyclin E and cyclin A) and Cdk1 (activated by cyclin A and cyclin B) [124,125]. Moreover, Cdt1 isolated from cell lysates co-precipitates cyclin A, but not cyclin E or cyclin B. CDK interaction depends on a cyclin binding motif known as a Cy motif in Cdt1 (aa 68–70) [125]. It is most likely that Thr29 phosphorylation to induce Cdt1 ubiquitylation by CRL1^Skp2^ is carried out by cyclin A/Cdk2 in early S phase, and Cdt1 phosphorylation in late S and G2 is carried out by cyclin A/Cdk1.

Interestingly, Thr29 can also be phosphorylated in vitro by the MAP kinase, Jnk1 (Jun kinase) [143]. Miotto and Struhl noted that treating cells to activate the stress kinase, Jnk1, also blocks Hbo1 recruitment to several selected origins [143]. Mutating Thr29 enhanced Hbo1 residence at origins, suggesting that Jnk1-mediated Cdt1 phosphorylation at Thr29 inhibits Hbo1 recruitment. Despite the ability of Jnk1 to phosphorylate Thr29, conditions that activate Jnk1 in cells (without causing DNA damage) had no effect on Cdt1 stability; the stability of Thr29-phosphorylated Cdt1 could indicate Jnk1-mediated inhibition of CRL1^Skp2^ or some other mechanism to prevent Cdt1 degradation [90]. In that study, the authors mapped multiple Cdt1 phosphorylation sites and determined which sites are sensitive to Jnk1 inhibitors. In addition to Thr29, at least two other phosphorylation sites showed the same Jnk1-sensitive pattern as Thr29: Ser93 and Ser318, but the outcomes of these phosphorylations have yet to be determined [143].

A concurrent study of Cdt1 phosphorylation by stress-activated MAP kinases focused on distinct sites in the linker domain and C-terminus. Chandrasekaran et al. mapped a collection of five sites that can be phosphorylated by either JNK or p38 MAPK isoforms: amino acids 372, 402, 406, 411 and 491 (Figure 2) [144]. Functional tests of phosphomimetic substitutions at these five positions led to the inference that Cdt1 phosphorylation not only inhibits its licensing activity in cells, but surprisingly, also blocks binding to the CRL4^Cdt2^ E3 ubiquitin ligase. As a result, this phospho-isoform of Cdt1 is resistant to degradation by CRL4^Cdt2^, though it is still sensitive to CRL1^Skp2^ [144,145]. The molecular mechanism of licensing inhibition is not yet known, but the concentration of phosphorylation sites in the linker domain hints that phosphorylation could affect the positioning of the central and C-terminal domains relative to one another. Another possibility is that the N- and C-termini are in close proximity to each other to allow phosphorylation in the linker to disrupt CRL4^Cdt2^ binding to the PIP degron.

Unlike Thr29 (and Ser31), these more C-terminally located phosphorylation positions are responsible for the commonly-observed mitotic Cdt1 gel mobility shift by sodium dodecyl sulphate-polyacrylamide gel electrophoresis (SDS-PAGE). This gel shift is evident not only in response to cellular stresses that activate p38 and JNK, but also during G2 and mitosis in unperturbed cell cycles and in quiescent cells [9,112,129,144]. Cdt1 is robustly phosphorylated in mitotic cells and dephosphorylated in early G1, though the phosphatase responsible is not known. Both p38 and JNK are active during mitosis alongside Cdk1 [146]. Since the phosphorylation sites match the consensus sequence for both classes of proline-directed kinases, it is currently impossible to know which kinase(s) are truly responsible for Cdt1 mitotic phosphorylation. Regardless of how many kinases target these sites, the result is that beginning in late S phase, Cdt1 re-accumulates in a form that is not active for origin licensing and is no longer subject to rapid degradation (Figure 3). A potential role for geminin in Cdt1 protection from CRL1^Skp2^ during G2 has also been reported [113,147], but attempts to definitively confirm this relationship have utilized geminin and Cdt1 manipulation which frequently induces DNA damage. Separating potential geminin-mediated direct effects on Cdt1 stability from established indirect effects related to re-replication dependent DNA damage (and subsequent CRL4^Cdt2^-mediated Cdt1 degradation) requires careful interpretation [148]. Nonetheless, the Cdt1 stabilization in G2 may serve the dual purposes of allowing Cdt1 to function with the NDC80 complex at kinetochores and providing a large pool of Cdt1 in the subsequent G1 to license origins in the next cell cycle.

Finally, the majority of detectable phosphorylation sites in human Cdt1 remain unstudied. Several of these match the consensus for CDK-mediated (or MAPK-mediated) phosphorylation. Are there additional CDK sites, and if so, are they dependent on the same Cy motif that directs phosphorylation at the N-terminus? Is there a MAPK docking site in Cdt1 that facilitates phosphorylation by p38 or JNK? The N-terminal PEST domain is in close proximity to several candidate sites which may function to inhibit Cdt1 chromatin binding in G2, though this idea has not been explicitly tested [112]. Of further note, all of the consequences of phosphorylation thus far lead to Cdt1 inhibition or changes in stability. It remains equally possible that phosphorylation at one or more novel sites promotes Cdt1 function in either licensing or mitosis.

## 5. Cdt1 in Disease

Although numerous mechanisms restrain Cdt1 function, pathological dysregulation of Cdt1 can still occur, particularly in cells whose control mechanisms have been compromised. Moreover, mutations in Cdt1 itself can cause pathological consequences.

### 5.1. Overexpression and Oncogenesis

Cdt1 overexpression can result in genotoxic stress leading to aberrant cell proliferation and predisposition to oncogenic transformation. Experimentally increased Cdt1 abundance outside of G1 phase promotes re-replication and genome instability [68,149,150]. Therefore, it is quite possible that more moderate overexpression from spontaneously deregulated transcriptional controls has the same effect on genome stability in vivo [7,151]. Over time, higher-than-normal levels of Cdt1 protein may not be fully restrained during S phase and G2 by the ubiquitin ligases, kinases, and geminin inhibition described in Section 4. This means that at some low frequency, Cdt1 may promote origin re-licensing and re-replication. Interestingly, cells expressing higher than normal Cdt1 exhibit a more aggressive and chemoresistant phenotype [152]. In addition, the genome instability from Cdt1 likely includes not only gene amplification and chromosome damage from re-replication [66], but also changes in chromosome number which may reflect Cdt1’s role in chromosome segregation [7,9].

Cdt1 transcription is driven by the E2F family of transcription factors (see Section 4.1), and one of the most frequently-mutated regulatory pathways in cancers is the Rb-E2F pathway [153,154]. In fact, many cancer-derived cell lines exhibit higher-than-normal expression of Cdt1 [117]. Cdt1 overexpression could also lead to rapid origin licensing and shorter G1, thus proliferate more rapidly but with less fidelity. To our knowledge, a direct and quantitative correlation between Cdt1 abundance and the extent of genome instability in cancers has not yet been investigated. It may be that Cdt1 expression levels will identify particular cancers that are most likely to progress or are more or less susceptible to specific therapeutic interventions [155].

### 5.2. Meier-Gorlin Syndrome

Avoiding either over- or under-licensing origins is critical for successful cell proliferation during development. The need for not only effective licensing inhibition after S phase but also efficient origin licensing in G1 is most apparent in the phenotypes associated with a rare primordial dwarfism syndrome, Meier-Gorlin Syndrome (MGS). MGS patients harbor hypomorphic mutations in genes encoding pre-RC components, including Cdt1, some ORC subunits, and Cdc6. These patients have extremely short stature, small external ears, and focal hypoplasias, likely due to slow cell proliferation [156,157,158]. Furthermore, *de novo* mutations in the gene encoding geminin, have also been described in MGS patients [159]. In these instances, the mutations disrupt protein motifs required for normal geminin degradation in G1 phase [159]. As a result, geminin could inappropriately inhibit Cdt1 and result in slow origin licensing and G1 delay. The Cdt1 MGS patient genotypes are compound heterozygous missense mutations combined with nonsense mutations (presumed null alleles) [156,158]. In addition, the *CDT1* mutations are present across most of the *CDT1* gene and translate to amino acid substitutions located in regions that are presumably important for Cdt1 regulation and activity. Cdt1 is an essential gene, so these alleles are likely hypomorphic rather than null.

Although origin licensing defects appear to be one molecular underpinning of MGS, not all MGS patients have mutations in origin licensing components. Recently, hypomorphic mutations in the *CDC45* gene that encodes one of the helicase activating subunits were identified in an additional cohort of MGS patients [160]. Cdc45 is not required for origin licensing in G1, but rather it is required for origin firing and replication fork progression during S-phase as part of the fully-active helicase (Cdc45-MCM2-7-GINS). These *CDC45* mutations result in splicing defects leading to a significant reduction in Cdc45 protein [160]. The change in Cdc45 protein abundance likely impairs DNA synthesis, thus hindering cell proliferation and genome stability in early development.

Interestingly, the pre-RC proteins affected in MGS include Cdt1, Cdc6, and subunits of ORC, but not MCM subunits. Do mutations in MCM lead to MGS phenotypes? Analyses of a spontaneous mutation in the mouse Mcm4 subunit suggests that dwarfism is not a universal outcome of licensing disruption. In these studies, hypomorphic Mcm4 mutations resulted in mice with increased micronuclei (a sign of chromosome instability) and increased tumor incidence, but otherwise grew to normal size [161]. Mouse embryonic fibroblasts from crosses between the hypomorphic and null alleles proliferate normally but are sensitive to replication stress [161]. These findings are in contrast to cells bearing MGS alleles in other licensing proteins that proliferate slowly [157,162]. These differences could reflect the degree of impairment by the different mutations, or they could reflect qualitative differences in the roles of the altered proteins. Such complexity certainly creates challenges for predicting the precise phenotypes of any newly-identified Cdt1 mutations, but the general expectation is impaired cell proliferation and/or genome instability.

## 6. Summary

Metazoan Cdt1 is regulated by a large number of independent mechanisms including inhibitor binding, phosphorylation, and ubiquitylation. It is likely that even more regulatory mechanisms will continue to be discovered, perhaps from follow-up studies to the proteomic detection of Cdt1 acetylation or sumoylation [104]. The need for such extensive regulation may be because Cdt1 is an integral player in both DNA replication and chromosome segregation, meaning Cdt1 deregulation has potent effects on genome stability and cell proliferation. The mitotic Cdt1 function clearly arose in eukaryotic evolution after the split between unicellular and multicellular species, so the presumed ancestral function was origin licensing. Why would Cdt1 evolve to have a role in kinetochore–microtubule attachment, a function that does not involve loading proteins onto DNA? It is becoming increasingly common to discover second cell cycle functions for origin licensing proteins, such as non-licensing roles for individual ORC subunits or geminin [139,140,141,163,164,165]. Perhaps it is generally useful to re-purpose proteins that are already under cell cycle-dependent control for a second cell cycle function. Alternatively, it may be that Cdt1 has biophysical properties that are uniquely suited to its molecular roles in both origin licensing and kinetochore–microtubule attachment. Based on our limited current knowledge, we can attempt to draw parallels between Cdt1’s two targets: the MCM and NDC80 complexes. In both cases, a multisubunit complex undergoes important conformational changes. For NDC80, the change manifests as a molecular extension in vivo, and for MCM it is the presumed opening and closing of the Mcm2–Mcm5 gate. It is thus tempting to speculate that Cdt1 stabilizes a particular (extended?) conformation in the MCM complex, and that its two cell cycle roles are in fact related. We look forward to future developments in the field that will continue to shed light on the regulation and function of this unique protein.
